# Epidemiological differences of common liver conditions between Asia and the West

**DOI:** 10.1002/jgh3.12275

**Published:** 2019-10-24

**Authors:** Thevaraajan Jayaraman, Yeong‐Yeh Lee, Wah‐Kheong Chan, Sanjiv Mahadeva

**Affiliations:** ^1^ Gastroenterology Unit, Faculty of Medicine Universiti Teknologi MARA Shah Alam Malaysia; ^2^ Department of Medicine, School of Medical Sciences Universiti Sains Malaysia George Town Malaysia; ^3^ Gastroenterology and Hepatology Unit, Faculty of Medicine University of Malaya Kuala Lumpur Malaysia

**Keywords:** acetaminophen toxicity, acute liver failure, acute‐on‐chronic liver failure, drug‐induced liver injury, epidemiology, hepatocellular carcinoma, non‐alcoholic fatty liver disease

## Abstract

Liver diseases form a heterogenous group of acute and chronic disorders of varying etiologies. Not only do they result in significant morbidity and mortality, but they also lead to a marked reduction in quality of life, together with a high socioeconomic burden globally. A better understanding of their global distribution is necessary to curb the massive health‐care and socioeconomic burden that they entail. Notable differences and similarities have been described between common liver disease conditions occurring in Asia and the West (Europe and North America), giving rise to the need for an updated collective appraisal of this subject. In this review, the epidemiological differences of common liver conditions, specifically acute liver failure, drug‐induced liver injury, acute‐on‐chronic liver failure, hepatocellular carcinoma, and non‐alcoholic fatty liver disease, between Asia and the West are discussed.

## Graphical abstract

The epidemiology of common liver conditions is different between Asia and the West. The predominance of viral hepatitis in Asia results in differences in etiology and prognosis of acute liver failure, acute‐on‐chronic liver failure, and hepatocellular carcinoma from that in the West. Etiological differences in drug‐induced liver injury (DILI) between Asia and the West may lead to a difference in prognosis

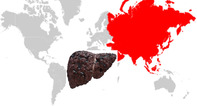

## Introduction

Liver diseases form a heterogenous group of acute and chronic disorders encompassing infectious, malignant, and inflammatory disease processes of varying etiologies. They confer a significant burden of disease, with liver cirrhosis alone contributing to 2% of all deaths globally in 2010.^1^ The Global Burden of Disease Study 2016 estimated that viral hepatitis, liver cancer, and liver cirrhosis caused 134 000, 830 000, and 1.3 million deaths, respectively, as observed using data pooled from 195 locations worldwide.^2^ The relative mortality rate for cirrhosis was found to be 27% higher compared to five different major cancers.^3^ In addition to the high mortality, there is also a high socioeconomic burden associated with liver diseases. In the United States, the annual cost of treating non‐alcoholic fatty liver disease (NAFLD) was projected to be US$103 billion.^4^ Hepatitis B‐related diseases were estimated to cost from 30 to 300% of the annual household income in China.^5^ Apart from the increased health‐care utilization and cost, people with chronic liver disease were less likely to be employed and had worse self‐reported general and mental health status.^6^

Although the burden of chronic liver disease and its sequelae, that is, cirrhosis and hepatocellular carcinoma (HCC), is increasingly recognized, geographical variations in epidemiology have been scarcely reported. The recognition of regional differences in the etiology of acute and chronic liver failure, for instance, have a profound effect on the clinical management of these conditions from one population to another. Diagnostic assessment and treatment algorithms for various liver diseases will differ depending on the recognition of these varied epidemiologies.

In this review, we aim to discuss the epidemiological differences of common liver conditions between Asia and the West, specifically acute liver failure (ALF), drug‐induced liver injury (DILI), acute‐on‐chronic liver failure (ACLF), HCC, and NAFLD. These liver conditions are well recognized globally, with certain epidemiological and clinical differences that will be highlighted in this review.

## Acute liver failure

ALF is a rare condition that can be broadly defined as acute and severe liver dysfunction causing coagulopathy and hepatic encephalopathy in a patient with no pre‐existing liver disorder. The specific definition of ALF is not standardized across studies, and there have been more than 40 variations of the definition found.^7^ In the United States, the incidence of ALF is 5.5 per million population, which yields an estimated 1600 new cases every year.^8^ ALF has a wide variety of causes that leads to significant heterogeneity in terms of outcome; for example, paracetamol‐related ALF is associated with the highest rate of recovery and lowest rate of death compared to other etiologies.^9^

The common causes of ALF in Asia and the West are shown in Table 1, while a comparison of the etiologies and outcomes between Asia and the West are outlined in Table 2. The predominant cause for ALF in the West is DILI, with paracetamol being the leading cause in the United States^15^, ^16^, ^20^ and the United Kingdom.^17^, ^18^ In some European countries, viral hepatitis remains a significant cause of ALF, with the most common etiology being hepatitis B.^19^, ^21^ In Asia, viral hepatitis is the predominant cause of ALF, but recent data indicate that DILI is increasingly common in Asia, apart from Japan.^11^ Unlike the West, however, the most common drugs implicated are herbal and traditional medications in China,^10^ whereas antituberculosis therapy is the main culprit in India.^14^ The major virus responsible for ALF in East Asia is hepatitis B, whereas in India, it is hepatitis E,^13^ where it is also endemic.

**Table 1:** Causes of acute liver failure and its survival outcome in Asia and the West

Author	Country	*n*	Viral hepatitis (%)	DILI (%)	Indeterminate cause (%)	Transplant‐free survival
Zhao^10^	China	177	11.3	43.5%: THR 16.9%	29.4	33%
Oketani^11^	Japan	460	46.1	14.6%	29.6	37.5%
Ho^12^	Taiwan	218	45.4	18.8%: PCM 11%	13.3	60%
Khuroo^13^	India	180	68.3	0.6%	31.1	31.3%
Kumar^14^	India	1223	43	ATT 7%	38	ATT 33%; HEV 54%; indeterminate cause 38%
Bower^8^	US	49	10	PCM 44.9%	NA	48.9%
Ostapowicz^15^	US	308	13	52%: PCM 39%; IDR 13%	17	43%
Reuben^16^	US	1198		11.1%	NA	27.1% (DILI only)
Marudanayagam^17^	UK	1237	3.7	68.1%: PCM 61.3%	15	71.1%
Bernal^18^	UK	2095		PCM 59.9%	NA	NA
Escorsell^19^	Spain	267	37	19.5%	32	43.6%

ATT, antituberculosis treatment; DILI, drug‐induced liver injury; HEV, hepatitis E virus; IDR, idiopathic drug reaction; PCM, paracetamol; THR, traditional herbal remedy.

**Table 2:** Comparison of etiology and outcome of acute liver failure between Asia and the West

Etiology	East (%)	West (%)
Viral	11.3–68.3	3.7–37
DILI	0.6–45.2	11.1–68.1
Indeterminate	11.9–38	15–32
Outcome		
Transplant‐free survival	31.3–60	27.1–71.1

DILI, drug‐induced liver injury.

Most studies in Asia and the West report similar rates of transplant‐free survival of approximately 30–50%, with notable exceptions being one study in the United Kingdom and another in Taiwan, both with reported rates of above 60%.

## Drug‐induced liver injury

The epidemiology of DILI is difficult to ascertain as it is often a diagnosis of exclusion, leading to an underestimation of the problem. Variations in biochemical patterns of liver injury and time to onset after drug exposure add to the challenge of recognizing DILI, thus contributing to underreporting.^22^ Reported incidence of DILI in the West varies from as low as 2.3–2.4 per 100 000 person‐years in the United Kingdom and Sweden,^23^, ^24^ to 14–19 per 100 000 person‐years in France and Iceland.^25^, ^26^ In contrast, the incidence of DILI in Asia appears to be higher based on a recent study from China with a reported annual incidence of 23.8 per 100 000 persons.^27^

Table 3 summarizes etiological differences in DILI between published reports from Asian populations and those in the West. The main culprits causing DILI in the West are antimicrobial agents and NSAIDs, with amoxycillin‐clavulanate and diclofenac being recognized as the most common drug in their respective categories. In contrast, the top causes for DILI in Asia are anti‐tuberculosis medications (particularly in India), traditional Chinese medications (especially in East and Southeast Asia), and other antimicrobial agents. Of interest, paracetamol overdose was only found to be a common cause for DILI in one study from Thailand.^38^ However, paracetamol hepatotoxicity is a recognized major cause for ALF, as alluded to in the previous section, and thus will be discussed further in the next section.

**Table 3:** Summary of studies on drug‐induced liver injury and the common causative agents

Author	Country	*n*	Study period	Common causes
Friis^28^	Denmark	1100	1978–1987	Halothane 25.5%, antimicrobials 15%, antiepileptic 9%
Sgro^25^	France	34	1997–2000	Antimicrobial 25%, psychotropic 22.5%, NSAIDs 10%
de Abajo^23^	UK	128	1994–1999	Amoxycillin‐clavulanate 10.2%, paracetamol 9.4%, diclofenac 7.8%
Andrade^29^	Spain	461	1994–2004	Amoxycillin‐clavulanate 12.8%, ebrotidine 5%, ATT 5%
Meier^30^	Switzerland	88	1996–2000	Heparin 37.5%, amoxycillin‐clavulanate 10.2%, NSAIDs 5.7%
De Valle^24^	Swedish	77	1995–2005	Diclofenac 18%, flucloxacillin 10.4%, azathioprine 6.5%
Björnsson3^31^	Iceland	96	2010–2011	Amoxycillin‐clavulanate 22%, diclofenac 6%, azathioprine 4%, infliximab 4%, nitrofurantoin 4%
Licata^32^	Italy	185	2000–2016	NSAIDs 35.5%, antibiotics 23.4%, immunosuppressants 10.9%
Chalasani^33^	US	300	2004–2007	Amoxycillin‐clavulanate 7.7%, nitrofurantoin 4.3%, isoniazid 4.3%, trimethoprim‐sulfamethoxazole 4.3%
Chalasani^34^	US	899	2004–2013	Amoxycillin‐clavulanate 10%, isoniazid 5.3%, nitrofurantoin 4.7%
Devarbhavi^35^	India	313	1997–2008	ATT 58%, antiepileptics 11%, olanzapine 5.4%
Rathi^36^	India	82	2014–2015	ATT 49%, antiepileptic 12%, CAM 10%
Wai^37^	Singapore	31	2004–2006	Traditional Chinese medications 55%, traditional Malay medications 16%, ATT 6%
Sobhonslidsuk^38^	Thailand	589	2009–2016	Paracetamol 35%, ATT 34.6%, antivirals 3.7%
Takikawa^39^	Japan	1676	1997–2006	Antibiotic 14.3%, neuropsychiatric drugs 10.1%, dietary supplements 10%
Aiso^40^	Japan	307	2010–2018	Anti‐inflammatory 11%, antimicrobial 11%, anticancer 10%
Suk^41^	South Korea	371	2005–2007	HM 27.5%, prescription medications 27.3%, health foods 13.7%
Kwon^42^	South Korea	567	2007–2008	ATT 19.8%, antiepileptics 9.7%, cephalosporins 9.5%
Zhu^43^	China	1985	2009–2014	Chinese HM 28.4%, antibiotics 10%, ATT 5%
Shen^27^	China	25 927	2012–2014	Traditional Chinese HM 26.8%, ATT 22%

ATT, antituberculosis medication; CAM, complementary and alternative medicine; HM, herbal medications; NSAID, non‐steroidal anti‐inflammatory drugs.

## Paracetamol‐induced hepatotoxicity

Paracetamol‐induced hepatotoxicity rates in Western Caucasian patients have been reported to be 15–36%.^44^, ^45^, ^46^ However, studies from Asia have reported paracetamol‐induced hepatotoxicity rates of only 2–7%.^47^, ^48^, ^49^ One possible explanation for this difference may relate to the quantity of paracetamol ingested during the overdose. The minimal amount of paracetamol reported to cause toxicity in adults is 7.5 g, and liver toxicity is typically associated with dosages of more than 10 g.^50^ Based on the published literature, patients with paracetamol‐induced hepatotoxicity in Asia seem to have ingested lower doses of the drug compared to Caucasian patients (Table 4), but the reason for this is uncertain.

**Table 4:** Summary of studies that have examined hepatotoxicity rates in patients with paracetamol overdose

Author	Country	Hepatotoxicity (%)	Survival (%)	Paracetamol dose
Schiødt, 1997^51^	USA	32	93	Median = 17.6 g 93% > 4 g
Hawton^52^	UK	31	NA	69% > 12.5 g
Gyamlani^53^	USA	16	98	NA
James^54^	USA	15 (1.3% ALF)	100	Mean = 18 g
Ayonrinde^45^	Australia	14	100	Median = 12 g
Mohd Zain^47^	Malaysia	7.3	100	38% > 10 g
Marzilawati^49^	Malaysia	7.5	100	Median 10 g (54.3% > 10 g)
Chan^48^	China	6	100	Median 5 g 6.7% > 10 g
Schmidt^55^	Denmark	No data on hepatotoxicity 0.9% (ALF)	99.9	Median 25 g

ALF, acute liver failure.

One explanation is that it could be due to differences in the rate of alcohol coingestion between Asia and the West. Chronic alcohol exposure is recognized to increase toxicity from paracetamol overdose by a two to threefold increase in hepatic content of cytochrome P4502E1, the major isoform responsible for the formation of the toxic metabolite from paracetamol.^56^ Regular and excessive alcohol consumption in Western patients with paracetamol overdose has been documented to be 25 and 20–40%, respectively.^49^ In contrast, the rate of alcohol coingestion was only 4.2–17% in Asian studies (Table 4).

Finally, variation in pharmacogenetics between Asians and Caucasians may explain the lower hepatotoxicity rates in the former. A study examining the excretion of paracetamol metabolites demonstrated heterogeneity in the conversion of paracetamol cysteine conjugates (toxic paracetamol metabolites) to mercapturate via N‐acetylation in healthy Chinese and Caucasian volunteers.^57^ Ethnic Chinese adults have been found to have relatively extensive glucuronidation but lower sulfation in paracetamol metabolism compared to Caucasians.

## Acute‐on‐chronic liver failure

ACLF is a clinical syndrome characterized by severe hepatic dysfunction following an acute insult in patients with underlying chronic liver disease or cirrhosis. In contrast to acute decompensated cirrhosis, ACLF has a high short‐term mortality, similar in prognosis to ALF.^58^ Currently, there is no single uniform definition for ACLF, and up to 13 different variations have been reported.^59^ The two most widely accepted definitions are given by the Asian Pacific Association for the Study of Liver (APASL) ACLF Research Consortium (AARC) and the European Association for the Study of Liver (EASL) Chronic Liver Failure Consortium (EASL‐CLIF).^60^, ^61^ While the AARC definition includes all patients with chronic liver disease, with or without cirrhosis, the EASL‐CLIF definition restricts itself to patients with cirrhosis only. This fundamental difference poses a significant challenge in making a reliable comparison of ACLF epidemiology between Asia and the West. The most common etiology of chronic insult in ACLF in Asia and the West is alcohol and viral hepatitis, with hepatitis B being the predominant virus in Asia and hepatitis C in Europe and North America (Table 5).^60^, ^62^, ^63^

**Table 5:** Underlying chronic liver disease in patients with acute‐on‐chronic liver failure

Region	Reference	*n*	Alcohol, (%)	Hep B, (%)	Hep C, (%)	Alcohol and Hep C, (%)	NASH, (%)	Others, (%)
Europe	60	303	60.3	0	13	9.3	0	17.4
North America	62	507	15	0	25	27	15.4	17.6
Asia Pacific	63	1402	56.1	15.1	1.9	0	6.1	20.8

Hep B, hepatitis B; hep C, hepatitis C; NASH, non‐alcoholic steatohepatitis.

The acute precipitating event for ACLF is also reported differently according to the EASL‐CLIF definition and the AARC definition. While the EASL‐CLIF criteria include both hepatic and nonhepatic insults, the AARC criteria accept only hepatic insults, thus making it difficult to make direct comparisons between the triggers for ACLF in Asia and the West.

Currently, the most common acute insult in Asia is alcohol (50.3%) followed by viral hepatitis (22.6%: hepatitis B; 13.2%, hepatitis E virus; 9.4%) and DILI (9.3%), and no attributable cause was found in 4.8% of cases.^63^ In Europe, 43.5% of ACLF cases have an unknown cause. Bacterial infections are the second most common trigger (32.6%) followed by alcohol (24.5%) and gastrointestinal hemorrhages (13.2%), with 13.5% of cases having more than one precipitating event.^60^

The acute insult for ACLF varies depending on geography and population studied and includes both infectious and noninfectious causes. The predominant acute insults triggering ACLF were previously reported to be quite distinct between Asia and the West. Infectious etiology was thought to predominate in Asia, while alcohol and DILI were the most common triggers in the West.^64^ However, alcohol has emerged as the major etiological agent more recently both in the West and Asia, with hepatitis B reactivation still being an important cause in Asia.^61^, ^63^ This is acknowledged by the AARC in their last consensus as being “a bit unexpected for the Asian countries” in relation to the rise of alcohol as a major acute hepatic insult, and this could be a result of the increasing “westernization” of Asia.^61^

The 28‐day and 90‐day mortality rate for ACLF in Asia was 40.5 and 49.2%, respectively, while it was 32.8 and 51.2% in Europe, respectively.^60^, ^63^

## Hepatocellular carcinoma

Primary liver cancer is the sixth most common cancer in the world, accounting for 5.6% of total cancers worldwide, and is ranked second for cancer mortality, causing 9.6% of cancer deaths in 2012.^65^ The predominant histological type of primary liver neoplasms globally is HCC.^66^ Asia harbors two‐thirds of the global HCC cases, with China having the highest incidence, with approximately 50% of new cases in 2012 occurring there. Table 6 highlights the major differences of HCC in Asia and the West. Asian men have a high incidence of primary liver cancer (namely HCC), with the highest incidence observed in East Asia and Southeast Asia.^65^ The incidence of HCC is much lower in North America and Europe. The overall incidence in women is lower compared to men, with Asian women having a higher incidence compared to women in Europe and North America.^65^ The mortality rates for HCC in Asia and the West follow an identical trend to the incidence rate, with a poor overall prognosis (ratio of mortality to incidence of nearly 1).^65^ The major differences in HCC between the West and Asia is summarized in Table 6.^65^, ^67^

**Table 6:** Differences in hepatocellular between Asia and the West

Differences	East	West
Incidence (age‐standardized rates per 100 000 persons)	East Asia: men; 31.9, women; 10.2 Southeast Asia: men; 22.2, women; 7.2 Decreasing incidence rate	Europe: men; 9.3, women; 2.2 North America: men; 6.8, women; 2.7 Increasing incidence rate
Etiology	HBV infection Aflatoxin	HCV infection Metabolic syndrome
Age of diagnosis	Younger age	Older age
Mortality (age‐standardized rates per 100 000 persons)	East Asia: men; 29.9, women; 9.6 Southeast Asia: men; 21.4, women; 6.8	Europe: men; 6.1, women; 2.2 North America: men; 6.8, women; 2.3

HBV, hepatitis B virus; HCV, hepatitis C virus.

HCC develops almost exclusively on the background of chronic liver disease, and in up to 90% of cases, the patient has liver cirrhosis.^68^ The dominant risk factors in East Asia (except Japan) and Southeast Asia is hepatitis B virus (HBV) infection and aflatoxin exposure, while in Europe and North America, it is hepatitis C virus (HCV) and the metabolic syndrome.^47^, ^50^ A strong geographic correlation has been demonstrated between the prevalence of HBV and the incidence of HCC: countries with high HBV infection prevalence (>8%) have a very high incidence of HCC.^66^ Exposure to aflatoxins, a recognized risk factor in Southeast Asian and East Asian countries, contributes to between 4.6 and 28.2% of all HCC cases worldwide.^69^ Aflatoxins have also been recognized to have synergistic effects with HBV in endemic areas to increase the risk of HCC.^70^

In Europe and the United States, 27–75% of HCC cases were attributed to HCV, while in Japan, it was 79% of cases.^71^, ^72^ The high prevalence of HCV‐associated HCC in Japan compared to the rest of East Asia is due to the mass utilization of used and unsterile hypodermic needles as part of a *schistosomiasis* eradication program in the 1950s.^73^ NAFLD is becoming an important risk factor as its incidence continues to rise, along with obesity and diabetes, worldwide. Data from the United States suggests that non‐alcoholic steatohepatitis (NASH) could be responsible for 59% of HCC, while in Japan, it was reported to be 2%.^74^, ^75^

Nevertheless, there has been a global change in the trend of HCC over recent times; normally high‐incidence countries in Asia are experiencing a decrease in new cases, while countries in North America and Northern Europe are reporting a rise in new cases.^67^, ^76^, ^77^, ^78^, ^79^ This has been attributed in part to the decreasing incidence of HBV due to national immunization programs and improved farming practices reducing the effect of aflatoxins in endemic countries, coupled with the unabated rise of metabolic syndrome and NAFLD in the developed world.

## Non‐alcoholic fatty liver disease

NAFLD is related to obesity and the metabolic syndrome and has been increasing in prevalence alongside the prevalence of obesity worldwide, particularly in Asia.^80^ A recent meta‐analysis showed that the prevalence of NAFLD in Asia, estimated at 27%, has now reached levels similar to that observed in the Western world.^81^

In both Asian and Western populations, NAFLD increases with increasing age, particularly between the ages of 30 and 50 years old, and is higher in men compared with women. The prevalence of NAFLD catches up in women after the age of 50 years, presumably due to the loss of the protective effect of female hormones. The prevalence of NAFLD also increases with increasing number of components of the metabolic syndrome. Hence, the screening for NAFLD is now considered in at‐risk groups, such as patients with diabetes mellitus and obesity, in both the Asian and Western guidelines.^82^, ^83^

Ethnic differences in the prevalence of significant hepatic steatosis has been reported in the West^84^; in a multiethnic population‐based study on 2287 subject in the United States using magnetic resonance spectroscopy, African Americans had a significantly lower prevalence of hepatic steatosis despite an equally high prevalence of obesity and insulin resistance compared with the Hispanics. In a separate study, the prevalence of cryptogenic cirrhosis (now recognized to be caused by NASH) was the highest among the Hispanics and the lowest among the African Americans despite the equally high prevalence of diabetes mellitus in the two ethnic groups.^85^ These observations were attributed to PNPLA3 gene polymorphism, which increased susceptibility to hepatic steatosis, which were more prevalent in Hispanics than in African Americans.^86^ Racial differences in the prevalence of NAFLD have also been observed in Asia. Several studies from multiethnic Malaysian populations consistently found the prevalence of NAFLD to be higher among the Malays and the Indians compared with the Chinese.^87^, ^88^, ^89^ The proportion of cryptogenic cirrhosis also mirrored the difference in the prevalence of NAFLD observed in the different ethnic groups, higher among the Malays and Indians at 21% compared with 12% among the Chinese.^90^ However, in a study on 198 healthy controls and 114 patients with biopsy‐proven NAFLD, the PNPLA3 gene polymorphism appeared to be higher among the Chinese compared with the Malays and Indians, suggesting that there could be differences in susceptibility to the gene polymorphism among the different Asian ethnic groups.^91^

NAFLD patients are at increased risk of mortality compared with the general population, with much of the mortality excess attributable to liver‐related complications in patients with NASH and advanced fibrosis.^92^ While longitudinal studies with paired liver biopsies have mostly come from Western populations,^93^ data from Asian populations are beginning to emerge.^94^, ^95^, ^96^ In two Asian studies comprising mostly NASH patients, fibrosis progression was observed in more than half of the patients over a median interval of 6 years.^94^, ^96^ Recent evidence points to fibrosis stage as the most important determinant of disease‐specific mortality in NAFLD.^97^

## Conclusion

This review of common acute and chronic liver conditions has highlighted several notable differences in epidemiology between Asia and the West. We have intentionally not discussed less common conditions such as autoimmune liver diseases, which are relatively infrequent, from a global perspective. Etiological differences in ALF and variation in paracetamol‐induced hepatoxicity between Asia and the West have clear implications on the clinical management of these conditions. Marked variations in DILI epidemiology between Asia and the West warrants further investigation into the factors underpinning the differences. Similarly, etiological and differing diagnostic criteria for ACLF between Asia and the West will influence the way this condition is detected and treated. Clinical variation in HCC between Asia and the West, largely due to epidemiological differences relating to viral hepatitis and NAFLD, will influence the prevention and treatment of this deadly disease. Finally, NAFLD is rising globally in tandem with the obesity and diabetes epidemic, with Asia having caught up to the West in terms of prevalence and disease burden.
